# Supplementary material for: Circulating microRNAs as a Prognostic Tool to Determine Treatment Efficacy in Lung Cancer Patients Undergoing Pembrolizumab PD-1 Blockade Immunotherapy
Source: Cancers (Basel). 2024 Dec 17;16(24):4202. doi: 10.3390/cancers16244202 (PMC11674096; doi:10.3390/cancers16244202)
Supplement: Supplementary file 1 [file cancers-16-04202-s001.zip › Supplementary Figures.pdf]

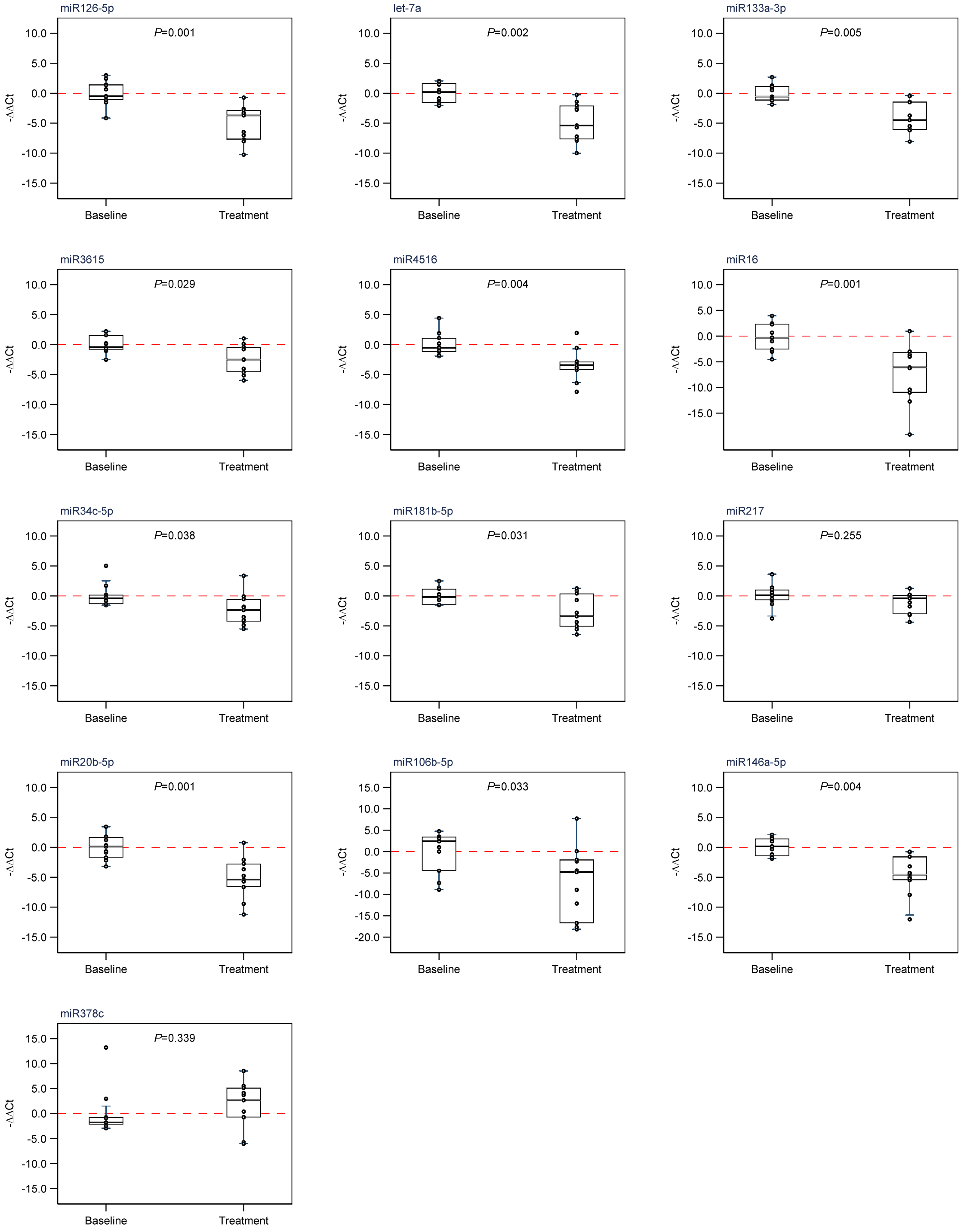

Supplementary Figure S1. Showing comparison of miRNA expression before and after treatment in female patients.

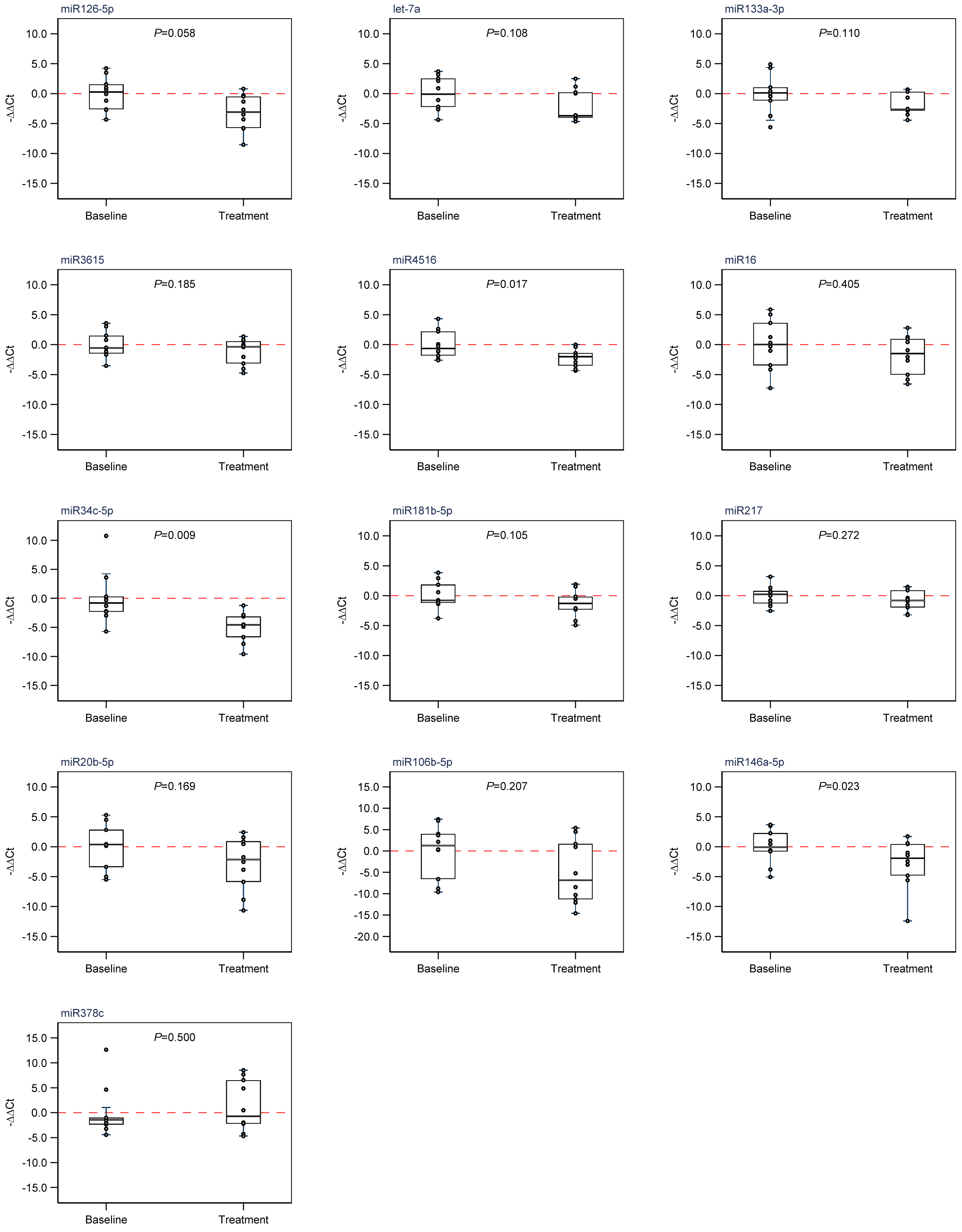

Supplementary Figure S2. Showing comparison of miRNA expression before and after treatment in male patients.

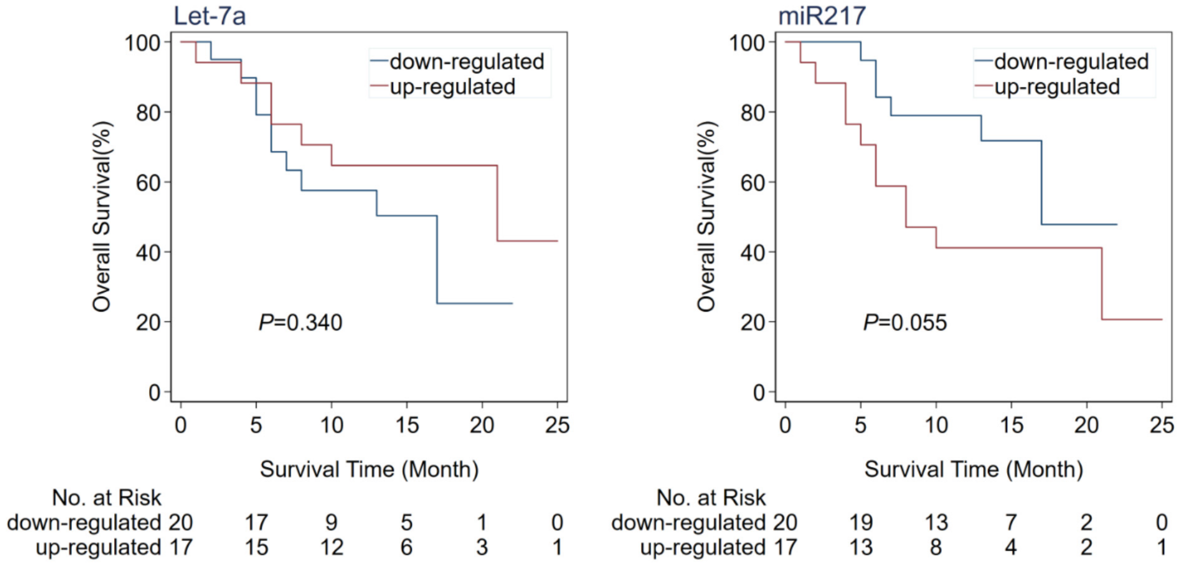

Supplementary Figure S3. The Kaplan Meier plots show patients' survival rates from diagnosis to the last contact. A) Initial let-7a levels; B) Initial miR217 levels.
